# Individual Choices of Wintering Areas Drive Adult Survival Heterogeneity in a Long‐Lived Seabird

**DOI:** 10.1002/ece3.70675

**Published:** 2024-12-12

**Authors:** M. Genovart, R. Ramos, J. M. Igual, A. Sanz‐Aguilar, G. Tavecchia, A. Rotger, T. Militão, D. Vicente‐Sastre, B. Garcia‐Urdangarin, R. Pradel, J. González‐Solís, D. Oro

**Affiliations:** ^1^ Theoretical and Computational Ecology Group CEAB (CSIC) Blanes Catalonia Spain; ^2^ Departament de Biologia Evolutiva, Ecologia i Ciències Ambientals, Facultat de Biologia Universitat de Barcelona (UB) Barcelona Spain; ^3^ Institut de Recerca de la Biodiversitat (IRBio) Universitat de Barcelona (UB) Barcelona Spain; ^4^ Animal Demography and Ecology Unit IMEDEA (CSIC‐UIB) Esporles Spain; ^5^ CNRS, EPHE, IRD CEFE, Univ Montpellier Montpellier France

**Keywords:** adult survival, *Calonectris diomedea*, hidden Markov models, migration, multievent mark‐recapture models, NAO, remote tracking, SOI

## Abstract

Seasonal migration has evolved as an adaptation for exploiting peaks of resource abundance and avoiding unfavourable climatic conditions. Differential migratory strategies and choices of wintering areas by long‐distance migratory species may impose varying selective pressures and mortality risks with fitness consequences. Recently developed tracking technologies allow wintering movements of migratory species to be studied. However, these technologies typically involve a limited number of tracked individuals, which gives low statistical power for any robust estimate of survival probabilities. Additionally, when utilising geolocators, data become accessible only upon individual recapture, presenting a potential source of bias. We used multievent modelling to include information of 147 identified wintering tracks in the analysis of 1104 long‐term individual capture histories (2000–2022) of migratory seabird *Calonectris diomedea* and then test if individual preferences for wintering areas may drive heterogeneity in adult survival. We also examined individual fidelity to wintering areas and tested if climatic and oceanographic conditions, as represented by the wNAO and SOI climatic indices, influenced survival and fidelity. The probability of fidelity to a wintering area was ca. 0.79. Annual changes between areas were influenced by environmental variability driven by the wNAO. Survival probability was influenced by the SOI and differed between wintering areas; these differences coupled with high wintering site fidelity, generated individual heterogeneity in adult survival. Our study reveals that, over the last two decades, some individuals wintered in less suitable areas, with nonnegligible consequences on adult survival, the parameter to which the population growth rate is most sensitive in long‐lived species. Winter oceanographic conditions such as stormy weather or the proximity to upwellings probably play a relevant role in driving survival heterogeneity. Further research is needed to enhance our understanding of how the interlinked effects of climate, local selective pressures and individual condition shape population dynamics in migratory species.

## Graphical abstract

Differential migratory strategies may impose varying selective pressures. While tracking technologies provide valuable information, they typically involve a limited number of individuals and may yield biased conclusions when data are only accessible upon individual recapture. We incorporated tracking information into a long‐term individual database and used Hidden Markov Models to examine wintering philopatry and to test whether individual preferences contribute to adult survival heterogeneity. Our study reveals that winter oceanographic conditions play a critical role in driving survival heterogeneity. Additionally, over the last two decades, some individuals have wintered in less suitable areas, with significant consequences for adult survival—a parameter to which the population growth rate is most sensitive in long‐lived species.

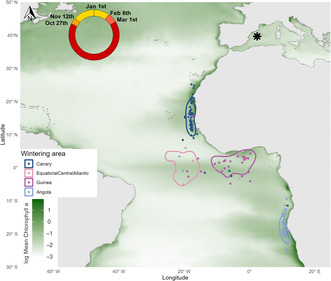

## 1 Introduction

Migration has evolved as a successful strategy in many organisms as a way of circumventing harsh climatic conditions and accessing more productive and safer environments that will help increase individual fitness (Greenberg and Marra 2005; Somveille et al. 2021). However, migratory animals have to cope with a variety of environmental conditions during their annual cycles, all of which may have an impact on adult survival (Newton 2024; Sergio et al. 2019; Sillett and Holmes 2002). Consequently, several studies have highlighted the importance of considering both breeding and wintering environmental conditions when assessing factors driving the population dynamics of migratory organisms (Greenberg and Marra 2005; Rushing, Ryder, and Marra 2016).

Previous studies of migratory top marine predators such as seabirds, seals and whales show that individuals typically migrate to areas where particular oceanographic features enhance marine productivity and food availability (Lesage et al. 2017; Pütz et al. 2006; Ream, Sterling, and Loughlin 2005). However, long‐distance movements to these areas can expose top marine predators to harsh weather conditions, thereby increasing mortality due to exhaustion, starvation or trauma caused by waves and strong winds (Hass, Hyman, and Semmens 2012; Newton 2024; Tavares et al. 2020).

Severe conditions at sea may vary not only annually but also geographically. Individuals from different populations generally have preferred, nonoverlapping wintering areas (Bogdanova et al. 2017; González‐Solís et al. 2007; Lagassé et al. 2022), although this may also be the case for individuals from the same population (Kürten et al. 2022). The choice of where to spend the winter and which migration route to take can entail differences in the balance between costs and benefits affecting individual fitness through immediate or lagged effects (Acker et al. 2021; Knudsen et al. 2011; Somveille et al. 2021). Long‐lived organisms such as pelagic seabirds are expected to show environmental canalisation (Gaillard and Yoccoz 2003; Gibson and Wagner 2000), keeping adult survival fairly constant despite environmental stochasticity. This entails that populations are severely affected when adult survival is reduced. Most demographic studies analyse variations in adult survival among breeding colonies or breeding areas, sometimes including individual differences in traits like sex, but seldom consider individual differences in winter distribution, when climatic conditions can be especially adverse. Understanding the demographic consequences of this heterogeneity in spatial winter distribution is thus crucial, as it may shed light on the ecological and evolutionary drivers underlying the evolution of migratory behaviours.

The Earth's climate undergoes oscillations. El Niño and La Niña are opposite phases of a natural climate pattern across the tropical Pacific Ocean that swings back and forth every 3–7 years on average (McPhaden, Zebiak, and Glantz 2006; Stenseth et al. 2003). Together, they are called ENSO which is short for El Niño‐Southern Oscillation. The ENSO cycle, measured by the Southern Oscillation Index (SOI), is the most influential natural climatic phenomenon on the Earth (Stenseth et al. 2003) and affects both the ocean and the atmosphere (McPhaden, Zebiak, and Glantz 2006). By disrupting the atmospheric circulation in the planet's largest oceanic basin, the phases of the ENSO shift the average location and strength of the mid‐latitude jet streams affecting temperature, rainfall and wind patterns across the tropics and generating also changes on the weather around the globe (NOAA, 2016; http://climate.gov). The North Atlantic Oscillation (NAO), is a large‐scale alternation of atmospheric mass between subtropical high surface pressure, centred on the Azores, and subpolar low surface pressures, centred on Iceland. The NAO is another influential climate phenomenon that modulates the strength and direction of westerly winds and location of storm tracks across the North Atlantic and also produces strong regional effects, especially in Western Europe (Hurrell et al. 2003; Hurrell and Deser 2009). Previous studies have shown that large‐scale climatic variation evaluated by climatic indices such as the SOI or the NAO influence population dynamics in many organisms (e.g., Coulson et al. 2001; Stenseth et al. 2002, 2003). Large‐scale climatic variation may be especially critical in the case of long‐distance migratory birds. For instance, Atlantic hurricanes and the trade wind intensities are modulated by the ENSO (Bell and Chelliah 2006; Klotzbach 2011; Roy and Reason 2001), which potentially influences the migration of marine animals such as seabirds to and from their wintering areas and thus is a potential driver of their survival probabilities. Additionally, climatic and oceanographic conditions may differ spatially, so individual wintering strategies may drive heterogeneity in mortality within a population of long‐lived seabirds.

Long‐term individual‐based demographic studies require significant dedication—in terms of both funding and time—but are crucial for describing the behavioural, ecological and evolutionary patterns of population dynamics (Clutton‐Brock and Sheldon 2010; Coulson 2020; Sheldon, Kruuk, and Alberts 2022; Tavecchia et al. 2017). While studies of population dynamics during the breeding period are commonplace, incorporating how winter environmental conditions affect migrating organisms that exploit different areas still remains a challenge. Tracking technologies may assist in this regard since they permit the study of the movements of elusive species such as seabirds and shed light on their migratory trajectories and wintering areas (Bernard et al. 2021; Burger and Shaffer 2008). Nevertheless, tracking has substantial economic, logistic and ethical costs, and most studies deal with a relatively small sample whose size alone hinders the obtaining of reliable estimates of individual survival, crucial for assessing individual fitness. Additionally, some tracking devices such as geolocators only provide information when recovered, a further possible source of bias in the data.

Scopoli's shearwater *Calonectris diomedea* is a long‐lived colonial seabird that breeds on Mediterranean islands and performs long‐distance annual migrations to the Atlantic Ocean off the west coast of Africa. Previous tracking studies have shown that individuals travel to subtropical areas in the southern Atlantic and that individual preferences for different wintering areas exist (González‐Solís et al. 2007; Reyes‐González et al. 2017). Demographic analysis based on individual‐based data collected at breeding colonies has found a temporal variation in adult survival associated with the large‐scale SOI index and noted that the NAO affects breeding success (e.g., Boano, Brichetti, and Foschi 2010; Genovart et al. 2013). Here, we use Hidden Markov Models (HMMs; McClintock et al. 2020; Pradel 2005) to combine long‐term individual capture‐recapture data of adult Scopoli's shearwater with tracking information from a subsample of individuals and evaluate whether or not preferences for wintering areas lead to adult survival heterogeneity in the population. We additionally assess fidelity to wintering areas and evaluate the role of a large‐scale climatic variation on these probabilities. This approach will allow us to increase the reliability of survival estimates and avoid the bias inherent in archival geolocator data. Even though the demography of pelagic seabirds is well studied globally, to our knowledge, this is the first time that the effects of individual wintering choices and climatic variation on adult survival in a population have been evaluated.

## 2 Material and Methods

### 2.1 Study Species and Study Colony

Scopoli's shearwater is a Procellariiform that breeds on Mediterranean islands and performs long‐distance migrations to tropical and trans‐equatorial waters in the Atlantic Ocean in October–March (González‐Solís et al. 2007). Most individuals arrive back in the Mediterranean by early March and breed from May to October (Ramos 2019; Reyes‐González and González‐Solís 2016). The species lays one egg and both members of the pair share incubation and chick‐feeding duties (del Hoyo, Elliott, and Sargatal 1992). It feeds mostly on fish, squid and crustaceans but also exploits fishing discards and suffers high bycatch mortality on longlines (Báez et al. 2014; Genovart et al. 2018).

Data were collected in 2000–2022 on Pantaleu, a rat‐ and carnivore‐free islet off the coast of Mallorca (39°34′ N, 2°21′ E, Balearic Archipelago) in the western Mediterranean, which holds a colony with ca. 200 breeding pairs of this shearwater (Sanz‐Aguilar, Igual, Oro et al. 2016). At this colony, breeding adults were captured and recaptured from mid‐May to mid‐July on nests.

### 2.2 Climatic and Oceanic Drivers

We used two global indices—SOI and NAO—to investigate the association between the demographic parameters of Scopoli's shearwaters and climatic variation. The SOI is a standardised index that measures the atmospheric component of a single large‐scale coupled interaction known as the ENSO. The ENSO is the most prominent known source of interannual variability in weather and climate and, although its effects are most patent in the South Pacific, it does in fact modify the weather throughout the globe (Stenseth et al. 2003). Global tropical cyclone activity in the North Atlantic is modulated by the ENSO, with more hurricane activity occurring in winter in positive SOI phases (i.e., La Niña) and less during the negative SOI phase (i.e., El Niño) (e.g., Holland 2009). In addition, Atlantic trade winds intensities are strongly modulated by the SOI (Bell and Chelliah 2006; Roy and Reason 2001). We used annual mean values for SOI (January–December), available at http://www.cru.uea.ac.uk/cru/data/soi/soi.dat, to investigate the association between climatic variation and annual survival.

The NAO index is based on the difference in the surface sea‐level pressure between the subtropical (Azores) high and the subpolar low regions (Hurrell 1995). Its variation influences ecological dynamics in both marine and terrestrial systems (Ottersen et al. 2001). We used the extended annual winter NAO—a good indicator of environmental conditions in the North Atlantic and Western Europe (Hurrell et al. 2003)—by averaging the winter (December–March) values (wNAO). Positive values of NAO give windy and warmer conditions in the North Atlantic and Western Europe, while the Mediterranean basin experiences drought. On the other hand, negative values of NAO result in colder winters in Western Europe and wetter conditions in the Mediterranean. We used the PC‐based wNAO index values, available at http://www.cgd.ucar.edu/cas/jhurrell/indices.html, to investigate the association between climatic variation and annual survival.

### 2.3 Tracking Data and Analyses

To determine the wintering areas of the shearwaters breeding on Pantaleu, 103 breeding Scopoli's shearwaters were fitted with a small, leg‐mounted, combined geolocator‐immersion logger (models Mk13 from British Antarctic Survey, Cambridge, UK, and Mk3005 [former Mk19] from Biotrack Ltd., Wareham, UK, weighing 1.5 and 2.5 g, respectively, corresponding to 0.2%–0.4% of their body mass). Every geolocator was calibrated before its deployment and after its recovery in a certain place with known coordinates, clear of shadows, and far from lit areas. To study fidelity to wintering area preferences, some birds were equipped with loggers for more than one season. Over the study period in 2002 and 2009–2014, we deployed a total of 258 geolocators. We recovered 182 geolocators, of which 147 provided useful data on the winter migration of 71 different individuals. Although previous studies have indicated that, when correctly deployed, these geolocators have negligible short‐term effects on birds (Carey, Meathrel, and May 2009; Igual et al. 2005), we re‐assessed the short‐term effects (first survival after deployment) and also the long‐term effects of the loggers on adult survival using capture‐recapture modelling (see Section 2.4).

Geolocators measured light intensity every minute, recording the maximum at 5‐ or 10‐min intervals depending on the geolocator model. We first determined the date and time of twilight events (i.e., sunrise and sunset) from raw light intensities using preprocessLight() and twilightEdit() functions of the *BAStag* package (Wotherspoon, Sumner, and Lisovski 2016). Second, we employed the *Solar/Satellite Geolocation for Animal Tracking (SGAT)* package (Lisovski et al. 2020), which uses Markov Chain Monte Carlo (MCMC) simulations to estimate and refine the locations of the tracked individuals. In our analysis, we undertook the following steps: (i) calculated the zenith angle and modelled the error distribution around the twilight times based on the calibration period of each geolocator at a known location, (ii) generated a gamma distribution of flight speeds from 0 to 40 km/h, reflecting the average values known for the species (Reyes‐González et al. 2017), (iii) used the thresholdPath() function to obtain the initial path of each bird, which is needed to begin the MCMC simulations and (iv) created a spatial mask to exclude locations over land and outside the range of the geolocators' raw data. By integrating all this information into the MCMC simulations, we obtained refined estimates of each bird's locations (see details in the *Tracking data analyses section* of the Appendix S1). This approach enhances the accuracy of location estimations, especially during equinox periods when latitude data is unreliable due to minimal variations in daylight length (Lisovski et al. 2012); nevertheless, some uncertainty may persist and the positional accuracy during the equinox periods remains lower than outside these periods (spatial accuracy of 304 ± 413 km according to Halpin et al. 2021). All geolocation data were processed using R 4.2.2 (R Core Team 2022).

**FIGURE 1:**
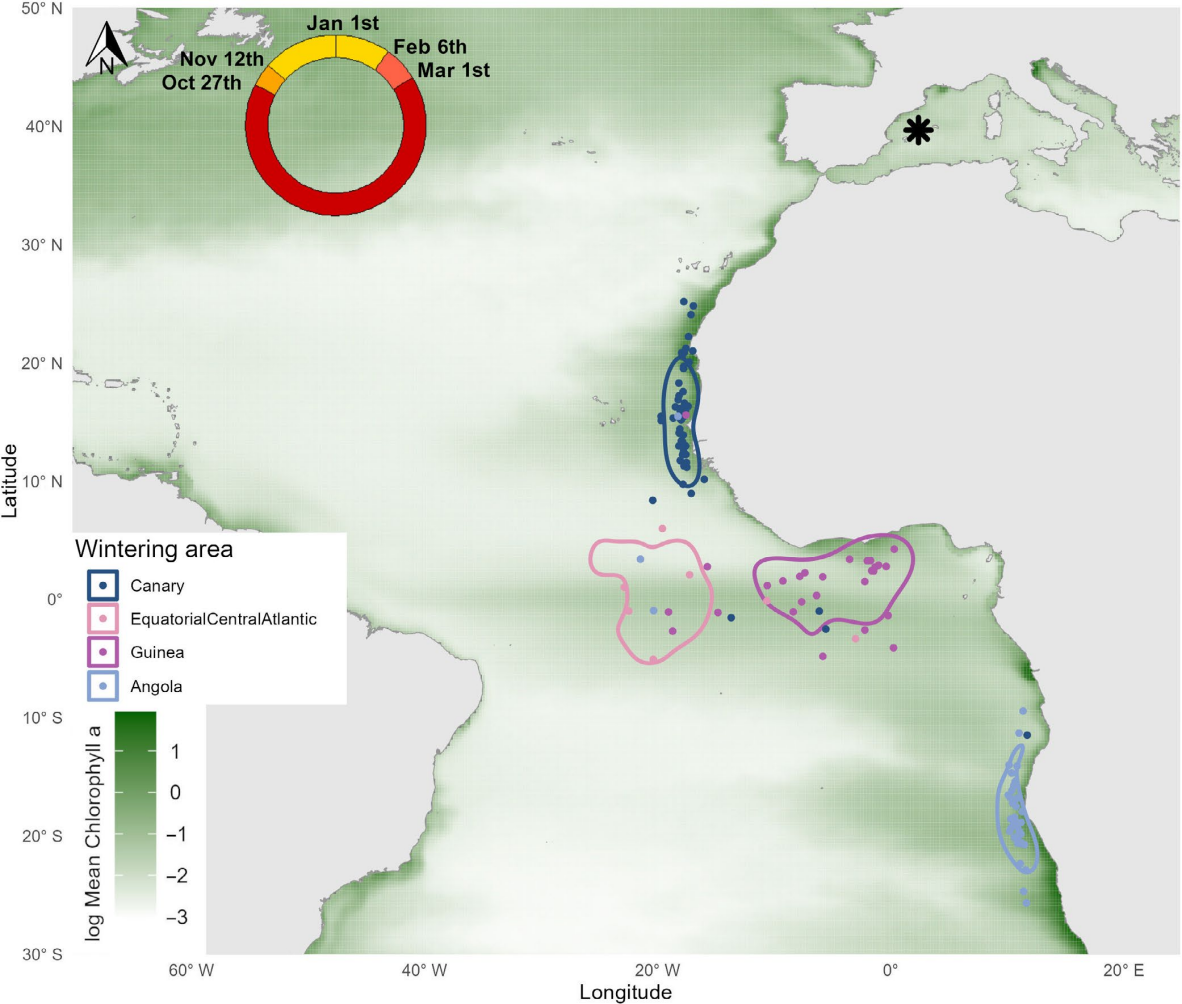
Main wintering areas of the Scopoli's shearwaters breeding on Pantaleu islet (Mallorca, Balearic Islands: 39°34′ 37″ N, 2°20′52″ E; marked with an asterisk). Each wintering area is depicted as a kernel density distribution (50% UDs): Dark blue for the Canary Current, light pink for the Equatorial Atlantic, magenta for the Gulf of Guinea, and light blue for the Angola‐Benguela Front. We show as dots on the map the individual annual wintering areas (i.e., centroids of wintering positions): 43.5% of the wintering registers were in the Canary Current (*n* = 64), 7.5% in the Equatorial Atlantic (*n* = 11), 21.8% in the Gulf of Guinea (*n* = 32) and 27.2% in Angola‐Benguela Front (*n* = 40). To visualise individual changes in wintering areas over the years, dots are coloured based on the most frequent wintering area for each individual, thus a few dots do not match the colour assignation of the kernel distributions. As an index of the regional oceanic productivity, we depicted in a green gradient the annual mean of surface Chlorophyll‐a concentration during the study periods (2002–2003 and 2009–2015, in log mg/m^3^; *Source*: Global ocean physics reprocessed models [L4] from www.marine.copernicus.eu, Copernicus Marine Service Information, EU). The upper schematic circle represents, clockwise, the annual phenology of all tracked shearwaters: Wintering period in yellow, breeding period in red, prebreeding migration in dark orange, and postbreeding migration in light orange. Estimated key migratory dates are also shown: Departure date from breeding area, first date in the wintering area, last date in wintering area and arrival date at the breeding site.

We ascribed the annual wintering areas of every individual track to one of four wintering areas: Canary Current, pelagic Equatorial Atlantic, Gulf of Guinea or Angola–Benguela Front, following Reyes‐González et al. (2017; Figure 1). Using estimated positions and the *move* R package (Kranstauber et al. 2012), we calculated the cumulative probability contours for specific kernel density Utilisation Distributions (UD): 5% UDs to estimate the centroids of the wintering ranges for every individual track and 50% UDs to estimate core areas of the habitat used in each wintering area (Figure 1; Lascelles et al. 2016). We used Krippendorff's alpha coefficient to estimate repeatability, that is, wintering site fidelity. This repeatability index ranges from 0, meaning that the same individual constantly changes its wintering area, to 1, meaning that the same individual always selects the same wintering area (Zango et al. 2019). We calculated that coefficient using the *krip.alpha()* function from the *irr* R package and performed 10,000 bootstrap iterations to obtain the 95% CI of the estimate (Gamer et al. 2019).

From the individualised year‐round tracks, we estimated four key migratory dates: (i) departure date from the breeding area, that is, the last day birds were present in the Mediterranean during their postbreeding migration period, (ii) their first day in their ascribed wintering area, that is, 50% UDs estimated for each wintering area, (iii) their last day in their ascribed wintering area and (iv) their arrival date at their breeding site, that is, back in the Mediterranean during the prebreeding migration period (Figure 1). All dates were first estimated using self‐designed routines written in R and then confirmed by visual inspection of the reconstructed tracks.

### 2.4 Demographic Data and Analyses

Each year, during the study period, we captured breeding adults and we marked unmarked birds with stainless‐steel rings with a unique code that allowed them to be identified in subsequent years. We then structured data for demographic analyses in individual encounter histories (Lebreton et al. 1992). In our study, wintering areas are only known for birds previously equipped with geolocators that have returned and been recaptured at the breeding colony (see Section 2.3). Additionally, the equipment is not permanent and an equipped bird may be released unequipped and vice versa. To keep track of these potential changes in equipment status, we split each yearly capture occasion into two: an ‘inter’ occasion reflecting the arrival state of the individual followed by an ‘intra’ occasion for the departure state. We used data from 2000 to 2022, that is, 23 annual encounter occasions, which could be translated into 45 coded encounter occasions. The ‘inter’ encounter occasions allowed us to test hypotheses regarding factors driving survival and migration decisions, while ‘intra’ occasions allowed us to record whether a geolocator had been deployed or not at the end of the season.

With this data set of encounter histories and before undertaking the demographic analysis, we first carried out a Goodness‐Of‐Fit (GOF) test using U‐CARE (Choquet et al. 2009) to assess the fit of our data to a general model. We tested the fit of the Cormack Jolly Seber (CJS) model, assuming full temporal variation in survival and recapture probabilities and considering only ‘inter’ occasions.

To analyse whether or not preferences for individual wintering areas drive annual survival probabilities, we used multievent capture‐recapture models (Pradel 2005) belonging to the HMMs family (McClintock et al. 2020). Models were fitted using the programme E‐SURGE (Choquet, Rouan, and Pradel 2009). These models had two levels: field observations, called ‘events’, encoded in the encounter histories, and ‘states’ reflecting the biological state of the individuals (which could only be inferred). With these models, we tested biological hypotheses accounting for changes between unobservable system states. We defined individual states based on wintering areas, trap heterogeneity (see below for explanations) and geolocator deployment (Appendix S1). Specifically, we defined four states for each wintering area: Canary Current, Gulf of Guinea, pelagic Equatorial Atlantic and Angola‐Benguela (Figure 1; see Section 2.3). From these four states, we differentiated those individuals without a geolocator, those with a fully functioning geolocator, and those with a geolocator that failed to provide data. For each of these states, we also defined two additional states, namely ‘aware’ and ‘unaware’ to incorporate trap‐dependence in the recapture probabilities as found by the GOF test (Pradel and Sanz‐Aguilar 2012). In our case, the GOF test showed that successive capture events were not independent and individuals detected on one occasion were more likely to be seen on a subsequent occasion. Finally, we defined an absorbing and unobservable ‘Dead’ state to account for mortality. In total, we considered 25 individual states (i.e., (4 × 3 × 2) + 1). For each individual state, we defined the corresponding possible events considering (i) whether or not the individual was captured at the colony, (ii) whether or not it was released with a geolocator and (iii) whether or not the geolocator provided information on the wintering area. Details on states, events and the multievent models can be found in Appendix S1.

Since our main goal was to assess whether or not differences in survival probabilities depend on the wintering area, we first developed several simple models to test this hypothesis (Models A). We subsequently added complexity to the models to investigate whether the survival during the first year after marking was different from subsequent years to estimate the probabilities that wintering areas change between years and to determine the factors driving these probabilities. Specifically, we tested the role of climatic variation in driving the abovementioned demographic parameters. We additionally tested whether geolocators impaired bird survival (Table 1). All our models included trap heterogeneity in the recapture probabilities; some models assumed variable recapture probabilities over time, while others assumed a constant recapture probability.

**TABLE 1:** Hypotheses tested to understand drivers affecting survival and wintering decision‐making in Scopoli's shearwater.

Biological hypotheses	
Use of wintering areas	Equal
	Area
	**Canary different from the others**
To decide on changing the wintering area	Constant
	To areas
	From areas
	Canary and others
	Canary, Equatorial, Angola
	Equatorial Atlantic, others
	**wNAO**
	SOI
	**Canary and others + wNAO**
	**Canary and others** * **wNAO**
Survival after first observed reproduction	**Equal from subsequent occasions**
	**Constant**
	Area
	wNAO
	SOI
Survival	Constant
	**Area**
	Canary, Equatorial, Angola
	Canary and others
	Equatorial Atlantic, others
	SOI
	NAO
	**SOI + area**
	SOI * area
	wNAO + area
	wNAO * area
	Canary, others + SOI
	Equatorial, others + SOI
	GEO

*Note:* Hypotheses supported by model selection (Table S1) are shown in bold (< 2 QAICc points from the best model). * and + indicate interaction and additivity, respectively. ‘Area’ means a different probability for individuals in each wintering area. ‘To area’ indicates a different probability of wintering in each different area in the subsequent year independent of the previous wintering areas. ‘From area’ indicates a different probability of changing the wintering area for birds wintering in each different area in the previous year. ‘wNAO’ indicates a different probability depending on the winter NAO values. ‘SOI’ indicates a different probability depending on the annual SOI values. ‘GEO’ indicates a different probability for individuals with and without a geolocator.

Models were compared using the Quasi Akaike Information Criterion corrected (QAICc) for the residual lack of fit by incorporating the variance inflation factor in its calculation (Lebreton et al. 1992). Additionally, we calculated the QAICc weight for each model as an index of its relative plausibility (Burnham and Anderson 2004).

## 3 Results

### 3.1 Migration

**FIGURE 2:**
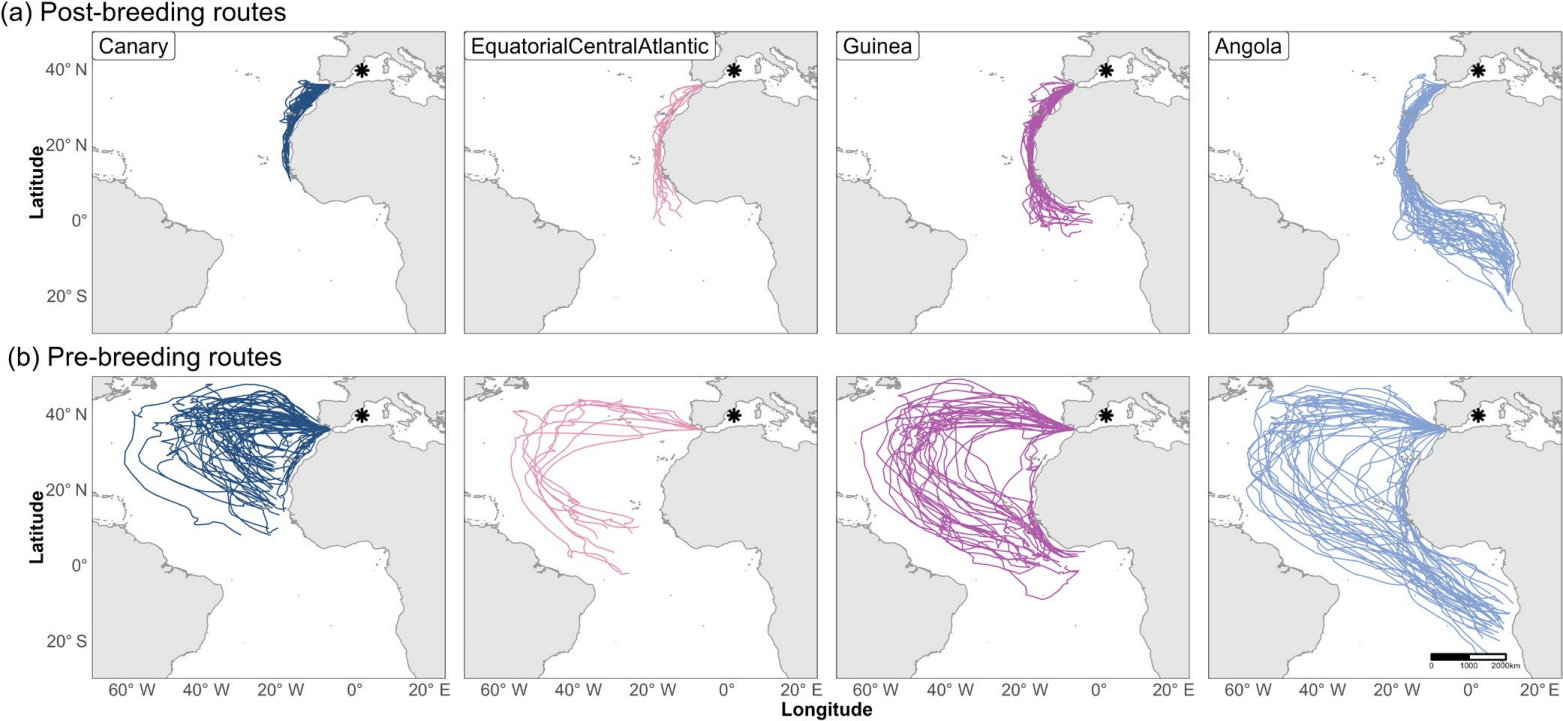
Individual migratory trajectories of Scopoli's shearwaters in the Atlantic Ocean. Individual (a) prebreeding and (b) postbreeding migratory trajectories are plotted as coloured lines depending on whether individuals wintered in the Canary Current (dark blue), the Equatorial Atlantic (light pink), the Gulf of Guinea (magenta) or the Angola‐Benguela Front (light blue). Trajectories are built with estimated positions from SGAT modelling (see Section 2). The breeding study colony is shown with an asterisk.

We obtained 148 migration tracks (Figure 2), which we used to define four nonoverlapping wintering areas (in parenthesis, the number of individual tracks): Canary Current (*n* = 64), Equatorial Atlantic (*n* = 11), Gulf of Guinea (*n* = 32) and Angola‐Benguela Front (*n* = 40; Figure 1). Wintering information for one individual who visited the Brazilian Current 1 year was not considered for the analysis. Of the 71 individuals with available tracks, 34 individuals provided information about one single migration cycle, 15 individuals about two migratory cycles, 11 individuals about three cycles, 4 individuals about four cycles, and 7 individuals about five cycles (Figure 1). Of the 37 individuals who were tracked more than once, 25 (67.5%) consistently visited the same wintering area, while 12 (32.5%) changed their wintering area at least once. This corresponded to Krippendorff's repeatability alpha coefficient of 0.639 (95% CI: 0.575–0.702).

The general annual phenology of all tracked shearwaters can be summarised as follows: postbreeding migration started on 27 October ±5.9 (17 October:14 November), with the arrival in the wintering areas on 11 November ±11.5 (22 October:16 December); prebreeding migration started on 06 February ±8.8 (08 January:25 February), with arrival back at the breeding area on 28 February ±7.7 (11 February:22 March; see schematic phenological circle in Figure 1). Postbreeding wintering routes for individuals going to southern trans‐equatorial wintering areas appeared to be more oceanic than those from individuals wintering in the Canary Current (Figure 2).

### 3.2 Demography

During 2000–2022, we registered a total of 23 annual encounter occasions, with 4295 observations of 1104 different individuals monitored while breeding on Pantaleu.

When analysing our data set, the GOF for the CJS model was very poor (*ĉ* = 6.631) mainly due to recapture heterogeneity (i.e., trap‐dependence, *χ* ^2^ = 459.944, df = 20). Based on this result, all our models included a trap‐dependent effect accounted for by differentiating the recapture probabilities of captured birds and birds missed on the previous occasion (Pradel and Sanz‐Aguilar 2012). We corrected for the remaining overdispersion using a variance inflation factor of 2.266.

**FIGURE 3:**
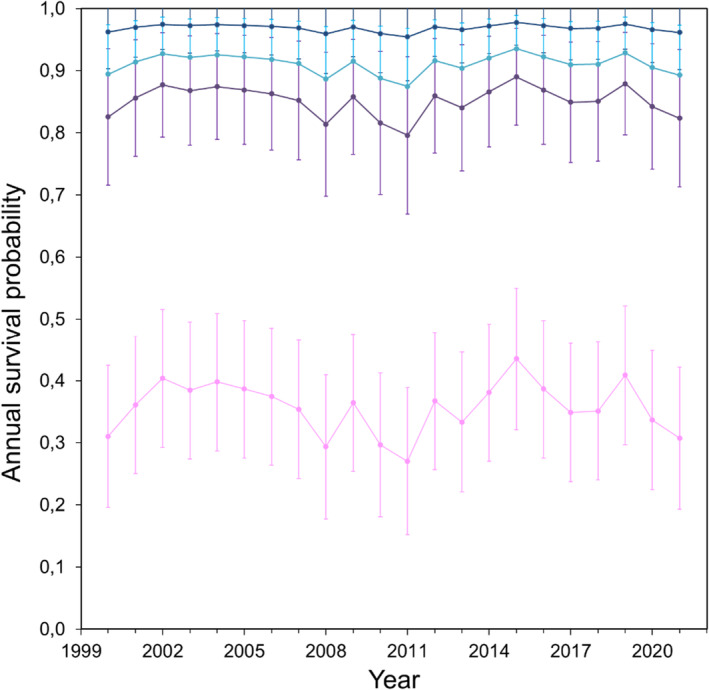
Variation in adult survival over the study period. The mean annual adult survival probability (± SE) estimated for individuals wintering in each area from the best model (Model 1, Table S1) assuming an additive effect of the SOI on adult survival in individuals wintering in each wintering area. Adult survival for individuals wintering in different areas are shown in different colours; Canary Current in dark blue, the Equatorial Atlantic in light pink, the Gulf of Guinea in magenta, and the Angola‐Benguela Front in light blue.

Our first models (Models A) clearly indicated that adult survival differed between individuals according to their wintering areas (Model A1, Table 2), with individuals wintering in the pelagic Equatorial Atlantic having the lowest survival probability (Table 3, Model A1). When adding more complexity to the models (Models B), some results should be taken with caution; six models were ranked at less than two points of QAICc (Table S1), so should be considered equally good at explaining the data, and many others, even if less plausible, were ranked also close. With these more complex models, we confirmed that survival varied according to the wintering area but also found that the SOI played an important role in driving annual survival probabilities (Tables 1 and 2, Table S2, Figure 3). All the six best‐ranked models considered that adult survival differed between wintering areas; five of the six also considered an effect of the SOI (Table S1). The survival probability was highest for individuals wintering in the Canary Current, followed by the Angola‐Benguela Front and the Gulf of Guinea, while those wintering in the pelagic Equatorial Atlantic had the lowest survival probability (Tables 1 and 2, Table S1,S2). Some of the best‐ranked models (Models 2 and 4; Table S1) suggest that the apparent survival probability is lower after the first encounter, probably following the first breeding attempt on Pantaleu. We estimated an increase by about 9% of mortality or permanent dispersal after the first breeding attempt (Table 1, Model 8 in Table S1,S2). Mean adult survival was 0.841 (0.821–0.859) (Table 3, Model 8, Table S1). We found no clear effect for loggers on adult apparent survival (Table S2) but cannot rule out a possible negative long‐term effect of geolocators (Table S2, M26 Table S1,S2).

**TABLE 2:** Model selection of the first simplest models to test the hypothesis that wintering areas affect adult survival in Scopoli's shearwaters on Pantaleu (Models A).

Model	Initial state	Survival	np	Deviance	QAICc	ΔAIC	AICw
A1	**Canary‐others**	**Area**	**33**	**9752.432**	**4362.494**	**0.000**	**0.815**
A2	Area	Area	35	9751.162	4365.967	3.474	0.144
A3	Canary‐others	Equal	30	9781.162	4369.104	6.610	0.030
A4	Area	Equal	32	9776.548	4371.101	8.608	0.011

*Note:* The selected model is shown in bold. ‘Initial state’ indicates wintering area location probabilities; ‘Area’ indicates a different survival probability for individuals in each wintering area; ‘Canary‐others’ indicates different probabilities for wintering in the Canary Current and all the other southern wintering sites; ‘Equal’ indicates equal probability; ‘np’ indicates the number of parameters; all models assumed a constant probability of changing wintering area (about 24%), a constant recapture probability that includes trap heterogeneity, and a time variant. QAICc indicates the AIC value corrected for overdispersion; ΔAIC indicates the QAICc difference points from the best model; AICw indicates the weight of each model *i*.

**TABLE 3:** Survival estimates for Scopoli's shearwater on Pantaleu.

Hypotheses		Survival	Mortality after first encounter	Model	ΔQAICc
Constant survival		0.827 (0.809–0.843)	—	A3	9.390
First survival different than subsequent		0.841 (0.821–0.859)	0.088 (0.045–0.165)	8	2.160
Wintering area	Canary	0.961 (0.414–0.999)		A1	2.780
	Guinea	0.821 (0.553–0.945)			
	Equatorial	0.428 (0.226–0.658)			
	Angola	0.903 (0.643–0.980)			
	Canary	0.969 (0.360–0.999)		5	1.070
	Guinea	0.850 (0.555–0.963)			
	Equatorial	0.362 (0.182–0.592)			
	Angola	0.911 (0.630–0.984)			

*Note:* Models used to estimate adult survival and assess whether or not there were differences in survival probabilities depending on the wintering area. Model A3 assumes constant adult survival regardless of the wintering area. Model 8 assumes different survival probabilities for individuals found at the colony for the first time (after the first observed reproduction) and constant survival for subsequent occasions, regardless of the wintering area. Model A1 assumes different survival probabilities depending on the wintering area. Model 5 also assumes different survival probabilities depending on wintering areas but the probability of changing the wintering area depends on the wNAO. For all estimates, the mean and the 95% confidence intervals are given. ΔQAICc indicates QAICc difference points from the best model (Table S1).

The annual probability of changing from one wintering area to another was 0.07 (0.038–0.131 95% CI; Model 8, Table S1). Although some shearwaters changed their wintering sites to one of the three other areas, about 79% of the individuals remained in the same area as in the previous winter. The six best selected models (< 2 QAICc points) showed that the individual decision to change the wintering area in consecutive seasons could be modulated by the wNAO (Table 1, Table S1). Our results are not conclusive as to whether the probability of changing the wintering area differs depending on the area (Table 1, Table S1). Model selection also indicated a slightly higher probability of wintering in the Canary Current area (0.28) than in any other wintering area (0.24 each; 0.166–0.336 95% CI) (Table 1, Table S1).

## 4 Discussion

By combining migration tracking with capture‐recapture data in an HMM framework, we revealed that individuals' preferences for different wintering areas lead to adult survival heterogeneities within the population in a long‐lived and long‐distance migratory seabird. We additionally confirmed that climatic conditions play a significant role in driving its population dynamics. On the one hand, SOI had a significant impact on the annual apparent survival probability. On the other hand, although individuals show fidelity to their wintering areas, they may occasionally change their wintering areas as a result of variable annual environmental conditions driven by the wNAO.

The demographic buffering hypothesis predicts selection for a reduction in the variance of the vital rates with the strongest influence on population growth and individual fitness (Hilde et al. 2020; Pfister 1998). In this context, in a long‐lived species such as Scopoli's shearwater, adult survival is expected to be fairly constant. Although some results showed overlapping confidence intervals, and further analyses are needed to fully understand the factors influencing adult survival in this species, we observed a nonnegligible variation in this vital rate—both among individuals wintering in different areas, and across years with varying climatic environmental conditions.

### 4.1 The Choice of Wintering Areas Drive Adult Survival Probabilities

We found that survival probabilities differ between wintering sites. Our results pointed to a higher mean adult survival in individuals wintering in the Canary Current and Angola and a critically low adult survival for those wintering in the pelagic Equatorial Atlantic. The strong upwelling in the Canary Current and the Angola‐Benguela Front, along with high primary productivity and the concentration of large marine predators (Jungblut et al. 2017; Scales et al. 2014) may be highly advantageous. However, we do not believe that this factor explains the low adult survival of birds wintering in equatorial areas, particularly in the pelagic Equatorial Atlantic. Additionally, the distance to the wintering area did not appear to play a significant role in driving the observed spatial heterogeneity in survival. We know that bycatch is currently affecting the adult survival of this species (Genovart et al. 2017, 2018), so one possibility could be a differential incidental bycatch in fisheries. However, we would expect higher bycatch incidence in the upwelling zones, where fishing activity is more intense. A possible explanation would be a higher incidence of captures in the equatorial zone if individuals with poorer capacities or compromised body conditions concentrate in this area (Dias, Granadeiro, and Catry 2012; Pardo et al. 2017). Age, sex and breeding experience may also play a role in explaining such differences if there is a spatial segregation in wintering areas driven by these individual traits (Chapman et al. 2011; Péron and Grémillet 2013; Sanz‐Aguilar et al. 2012). A previous study analysing migration strategies of this species in Linosa (Italy) found extensive sex differences in the scheduling, duration, distances and destinations of migratory routes (Müller et al. 2014). However, another study based on data from our study colony failed to find significant differences in the use of wintering areas between males and females, although it did detect a slight tendency for females to visit the Guinea Gulf area more often than males (De Felipe et al. 2019). Another previous study showed similar survival between females and males in our study colony (Choquet et al. 2013).

### 4.2 The Role of Climatic Conditions in Driving Adult Survival

As previously found, the large‐scale ENSO cycle, tracked by the SOI, seems to play a relevant role in driving adult survival in this and other long‐lived marine species (Genovart et al. 2013; Ramos et al. 2012; Tavares et al. 2020). The mechanism through which ENSO influences adult survival in this species remains unknown, though one possibility is a direct impact from extreme weather conditions. A recent study found that oceanic seabirds chase tropical cyclones (Ventura et al. 2024). Extreme weather conditions such as hurricanes, generate intense surface ocean cooling and vertical mixing, resulting in nutrient upwelling into the photic zone and episodic phytoplankton blooms (Fiedler et al. 2013; Pedrosa‐Pàmies et al. 2019). One possibility is that hurricanes may offer predictably favourable wind conditions and foraging opportunities in most years (Ventura et al. 2024) while negatively impacting adult survival during years with particularly extreme adverse conditions.

The role of ENSO‐driven climatic conditions also seems to differ between wintering areas. The weaker effect of the SOI in the Canary Current compared to regions further south may be explained by the increased susceptibility to variations in the SOI in the Southern Hemisphere (McPhaden, Zebiak, and Glantz 2006; Yang et al. 2018). Additionally, individuals wintering in southern areas seem to use more oceanic routes, which exposes them to more severe stormy weather than birds wintering in the Northern Hemisphere (Morera‐Pujol et al. 2023; Reyes‐González et al. 2017). However, individuals wintering in the most southerly area (Angola) did not have the lowest survival rate, so other factors beyond the SOI are probably driving variations in survival probabilities between wintering sites (see also previous section).

A previous study of this species found that the SOI affected adult survival differently in two separate Mediterranean colonies (up to 66% in Pantaleu and 26% of variance explained in Chafarinas; Genovart et al. 2013). In light of our results, the previous finding could be explained by differences in the choice of wintering areas in individuals from these two colonies. This would imply that possible future variations in climatic conditions would differentially affect individuals in the same colony and also the population dynamics of different colonies.

### 4.3 The Role of Climatic Conditions in Driving Migratory Strategies

Our study further supports the idea that the probability of changing the wintering area from 1 year to another is low, approximately 0.20 (see also De Felipe et al. 2019), and that environmental conditions driven by the wNAO influence this probability. The wNAO is a good indicator of environmental conditions in the North Atlantic and the European continent (Hurrell et al. 2003), which may suggest that environmental conditions at sites while breeding, also influence migratory strategies. These results agree with previous studies that show that individual body condition after reproduction affects subsequent migration (Catry et al. 2013). While individuals show high fidelity to wintering sites, our results also indicate some plasticity in their migratory strategies, which clearly benefits the species' adaptation to changing climatic conditions.

### 4.4 Conservation Concerns

Due to the low adult survival, Pantaleu is, at the moment, not self‐maintained but rescued by immigration (Sanz‐Aguilar, Igual, Oro et al. 2016; Sanz‐Aguilar, Igual, Tavecchia et al. 2016; Tenan et al. 2014). Even if this colony seems more affected by climatic conditions than other colonies, and some wintering strategies seem not to be adaptative, with the available information, we would not consider this a threat to the species or the colony. The species has evolved to cope with environmental changes, and the observed variability in migratory strategies both within individuals and among colonies is also reassuring. At the moment, we consider that the main concerns for the conservation of most pelagic seabirds are accidental bycatch in fishing gears and the presence of terrestrial predators in the colonies.

## 5 Conclusions

We show here that climate and wintering strategies play a significant role in driving adult survival in a long‐lived seabird that undertakes long‐range migrations. However, additional studies with more information on wintering strategies are needed for gaining a deeper understanding of the factors determining wintering spatial segregation and its role in driving the population dynamics of this species. The use of different wintering areas entails associated costs and advantages, with individual decisions likely to be influenced by environmental conditions such as climate, biotic factors including density‐dependence and food availability, and by individual experience or physical condition. Nevertheless, it remains unclear whether the effects of the SOI on adult survival in this long‐lived species match or exceed past patterns, nor what the population‐level consequences might be of the increasing frequency and intensity of stormy weather in tropical regions provoked by the increasing number of extreme climate events. Future studies combining tracking and individual demographic data should help disentangle the complex interplay between climate, individual traits and migratory decision‐making that drives population dynamics in migrant animal populations.

## Author Contributions

**M. Genovart:** conceptualization (equal), data curation (equal), formal analysis (lead), funding acquisition (equal), writing – original draft (lead). **R. Ramos:** conceptualization (equal), formal analysis (equal), datacuration (equal), visualization (lead), writing – review and editing (equal). **J. M. Igual:** conceptualization (equal), data curation (lead), investigation (equal), writing – review and editing (equal). **A. Sanz‐Aguilar:** conceptualization (equal), investigation (equal), writing – review and editing (equal). **G. Tavecchia:** conceptualization (equal), funding acquisition (equal), investigation (equal), writing – review and editing (equal). **A. Rotger:** investigation (equal), writing – review and editing (equal). **T. Militão:** formal analysis (equal), data curation (equal), writing – review and editing (equal). **D. Vicente‐Sastre:** formal analysis (equal), visualization (equal), writing – review and editing (equal). **B. Garcia‐Urdangarin:** formal analysis (equal), visualization (equal), writing – review and editing (equal). **R. Pradel:** conceptualization (equal), formal analysis (equal), writing – review and editing (equal). **J. González‐Solís:** conceptualization (equal), formal analysis (equal), funding acquisition (equal), writing – review and editing (equal). **D. Oro:** conceptualization (equal), data curation (equal), funding acquisition (equal), investigation (equal), writing – review and editing (equal).

## Conflicts of Interest

The authors declare no conflicts of interest.

## Supporting information


Appendix S1

Table S1‐S2

## Data Availability

Data availability and some codes to reproduce our tracking analyses are available via the CSIC and CORA repositories: https://doi.org/10.20350/digitalCSIC/16349 and https://doi.org/10.34810/data1864.
