# Supplementary material for: Individual Choices of Wintering Areas Drive Adult Survival Heterogeneity in a Long‐Lived Seabird
Source: Ecol Evol. 2024 Dec 12;14(12):e70675. doi: 10.1002/ece3.70675 (PMC11638144; doi:10.1002/ece3.70675)
Supplement: Supplementary file 1 — Appendix S1 [file ECE3-14-e70675-s001.pdf]

Individual choices for wintering areas drive heterogeneity in adult survival in a long-lived seabird

M Genovart, R Ramos, JM Igual, A Sanz-Aguilar, G Tavecchia, A Rotger, T Militão, D Vicente, B Garcia-Urdangarin, R Pradel, J Gonzalez-Solís, D Oro

Appendix. Specification of the multi-event modeling approach

Multi-event models were built using program E-SURGE (Choquet et al. 2009). We first describe the states and events defined in our modelling approach and then specify the steps and specific GEMACO syntaxis. We define an initial state vector and the transition and encounter matrices. Transitions between states were modeled in a six-step approach: 1) Geolocator functioning, 2) Changing wintering location, 3) Survival of the first encounter, 4) Survival after the first encounter 5) recapture probability, 6) Geolocator placement.Each step represents the different parameters to estimate. This is done by means of row-stochastic matrices, i.e. each row corresponds to a multinomial. Consequently, the total of cell probabilities is 1. Because of this constraint, one and only one cell probability in each row will be calculated as the complement to 1 of the others. This particular cell is denoted with a '*' symbol. Inactive cells, i.e. cells whose associated probability is structurally 0 are denoted with a '-' symbol. An active cell receives an arbitrary letter. Note that the same letter in two cells does not mean that the two values should be equal. We only show formula of the best Model (Model 1, Table S1)

States

We define 25 diferent states.

CNA: Canary previous winter/No Geo/Aware
CNU: Canary previous winter/No Geo/Unaware
CFA: Canary previous winter/Failed Geo/Aware
CFU: Canary previous winter/Failed Geo/Unaware
CGA: Canary previous winter/Geo/Aware
CGU: Canary previous winter/Geo/Unaware

GNA: Guinea previous winter/No Geo/Aware
GNU: Guinea previous winter/No Geo/Unaware
GFA: Guinea previous winter/Failed Geo/Aware
GFU: Guinea previous winter/Failed Geo/Unaware
GGA: Guinea previous winter/Geo/Aware
GGU: Guinea previous winter/Geo/Unaware

SNA: At sea Guinea previous winter/No Geo/Aware

SNU: At sea Guinea previous winter/No Geo/Unaware
SFA: At sea Guinea previous winter/Failed Geo/Aware
SFU: At sea Guinea previous winter/Failed Geo/Unaware
SGA: At sea Guinea previous winter/Geo/Aware
SGU: At sea Guinea previous winter/Geo/Unaware

NNA: Namiby-Angola previous winter/No Geo/Aware
NNU: Namiby-Angola previous winter/No Geo/Unaware
NFA: Namiby-Angola previous winter/Failed Geo/Aware
NFU: Namiby-Angola previous winter/Failed Geo/Unaware
NGA: Namiby-Angola previous winter/Geo/Aware
NGU: Namiby-Angola previous winter/Geo/Unaware

D: Dead

Events

Each occassion is decomposed by two event codes. One for encounter occasions "intra" and one for ocassions "inter".

Events "Inter" occasions

0 Not seen. Wintering Unknown
1 Seen alive. Wintering Unknown
2 Seen alive. Wintering Canary
3 Seen alive. Wintering Guinea
4 Seen alive. Wintering at sea at Equatorial Guinea
5 Seen alive. Wintering at sea at Namibia-Angola
6 Not seen. Wintering Canary
7 Not seen. Wintering Namibia-Angola
We have no observations of individuals not seen but wintering in Equatorial Guinea and Guinea

Events "Intra" occasions

0 Nothing change
1 Depart without GEO
2 Depart with GEO

Shortcuts

In E-surge syntax, and for the sake of simplicity, we define several shortcuts:
inter : t(2:2:46)
intrao: t(2:2:46)

intero: t(1:2:46)
area: from(1:5,7:12,13:18,19:24)
toFA : to(3:6:25)
toGA : to(5:6:25)

Initial States

CNA	CNU	CFA	CFU	CGA	CGU	GNA	GNU	GFA	GFU	GGA	GGU	SNA	SNU	SFA	SFU	SGA	SGU	NNA	NNU	NFA	NFU	NGA	NGU
*	−	−	−	−	−	i	−	−	−	−	−	i	−	−	−	−	−	i	−	−	−	−	−

GEMACO formula: i

Transition matrices

1 Geo functioning

	CNA	CNU	CFA	CFU	CGA	CGU	GNA	GNU	GFA	GFU	GGA	GGU	SNA	SNU	SFA	SFU	SGA	SGU	NNA	NNU	NFA	NFU	NGA	NGU	D
CNA	*	−	−	−	−	−	−	−	−	−	−	−	−	−	−	−	−	−	−	−	−	−	−	−	−
CNU	−	*	−	−	−	−	−	−	−	−	−	−	−	−	−	−	−	−	−	−	−	−	−	−	−
CFA	−	−	*	−	−	−	−	−	−	−	−	−	−	−	−	−	−	−	−	−	−	−	−	−	−
CFU	−	−	−	*	−	−	−	−	−	−	−	−	−	−	−	−	−	−	−	−	−	−	−	−	−
CGA	−	−	f	−	*	−	−	−	−	−	−	−	−	−	−	−	−	−	−	−	−	−	−	−	−
CGU	−	−	−	f	−	*	−	−	−	−	−	−	−	−	−	−	−	−	−	−	−	−	−	−	−
GNA	−	−	−	−	−	−	*	−	−	−	−	−	−	−	−	−	−	−	−	−	−	−	−	−	−
GNU	−	−	−	−	−	−	−	*	−	−	−	−	−	−	−	−	−	−	−	−	−	−	−	−	−
GFA	−	−	−	−	−	−	−	−	*	−	−	−	−	−	−	−	−	−	−	−	−	−	−	−	−
GFU	−	−	−	−	−	−	−	−	−	*	−	−	−	−	−	−	−	−	−	−	−	−	−	−	−
GGA	−	−	−	−	−	−	−	−	f	−	*	−	−	−	−	−	−	−	−	−	−	−	−	−	−
GGU	−	−	−	−	−	−	−	−	−	f	−	*	−	−	−	−	−	−	−	−	−	−	−	−	−
SNA	−	−	−	−	−	−	−	−	−	−	−	−	*	−	−	−	−	−	−	−	−	−	−	−	−
SNU	−	−	−	−	−	−	−	−	−	−	−	−	−	*	−	−	−	−	−	−	−	−	−	−	−
SFA	−	−	−	−	−	−	−	−	−	−	−	−	−	−	*	−	−	−	−	−	−	−	−	−	−
SFU	−	−	−	−	−	−	−	−	−	−	−	−	−	−	−	*	−	−	−	−	−	−	−	−	−
SGA	−	−	−	−	−	−	−	−	−	−	−	−	−	−	f	−	*	−	−	−	−	−	−	−	−
SGU	−	−	−	−	−	−	−	−	−	−	−	−	−	−	−	f	−	*	−	−	−	−	−	−	−
NNA	−	−	−	−	−	−	−	−	−	−	−	−	−	−	−	−	−	−	*	−	−	−	−	−	−
NNU	−	−	−	−	−	−	−	−	−	−	−	−	−	−	−	−	−	−	−	*	−	−	−	−	−
NFA	−	−	−	−	−	−	−	−	−	−	−	−	−	−	−	−	−	−	−	−	*	−	−	−	−
NFU	−	−	−	−	−	−	−	−	−	−	−	−	−	−	−	−	−	−	−	−	−	*	−	−	−
NGA	−	−	−	−	−	−	−	−	−	−	−	−	−	−	−	−	−	−	−	−	f	−	*	−	−
NGU	−	−	−	−	−	−	−	−	−	−	−	−	−	−	−	−	−	−	−	−	−	f	−	*	−
D	−	−	−	−	−	−	−	−	−	−	−	−	−	−	−	−	−	−	−	−	−	−	−	−	*

GEMACO formula: intra+inter
Fixed value 0 for intra

2 Wintering

	CNA	CNU	CFA	CFU	CGA	CGU	GNA	GNU	GFA	GFU	GGA	GGU	SNA	SNU	SFA	SFU	SGA	SGU	NNA	NNU	NFA	NFU	NGA	NGU	D
CNA	*	–	–	–	–	–	w	–	–	–	–	–	w	–	–	–	–	–	w	–	–	–	–	–	–
CNU	–	*	–	–	–	–	–	w	–	–	–	–	–	w	–	–	–	–	–	w	–	–	–	–	–
CFA	–	–	*	–	–	–	–	–	w	–	–	–	–	–	w	–	–	–	–	–	w	–	–	–	–
CFU	–	–	–	*	–	–	–	–	–	w	–	–	–	–	–	w	–	–	–	–	–	w	–	–	–
CGA	–	–	–	–	*	–	–	–	–	–	w	–	–	–	–	–	w	–	–	–	–	–	w	–	–
CGU	–	–	–	–	–	*	–	–	–	–	–	w	–	–	–	–	–	w	–	–	–	–	–	w	–
GNA	w	–	–	–	–	–	*	–	–	–	–	–	w	–	–	–	–	–	w	–	–	–	–	–	–
GNU	–	w	–	–	–	–	–	*	–	–	–	–	–	w	–	–	–	–	–	w	–	–	–	–	–
GFA	–	–	w	–	–	–	–	–	*	–	–	–	–	–	w	–	–	–	–	–	w	–	–	–	–
GFU	–	–	–	w	–	–	–	–	–	*	–	–	–	–	–	w	–	–	–	–	–	w	–	–	–
GGA	–	–	–	–	w	–	–	–	–	–	*	–	–	–	–	–	w	–	–	–	–	–	w	–	–
GGU	–	–	–	–	–	w	–	–	–	–	–	*	–	–	–	–	–	w	–	–	–	–	–	w	–
SNA	w	–	–	–	–	–	w	–	–	–	–	–	*	–	–	–	–	–	w	–	–	–	–	–	–
SNU	–	w	–	–	–	–	–	w	–	–	–	–	–	*	–	–	–	–	–	w	–	–	–	–	–
SFA	–	–	w	–	–	–	–	–	w	–	–	–	–	–	*	–	–	–	–	–	w	–	–	–	–
SFU	–	–	–	w	–	–	–	–	–	w	–	–	–	–	–	*	–	–	–	–	–	w	–	–	–
SGA	–	–	–	–	w	–	–	–	–	–	w	–	–	–	–	–	*	–	–	–	–	–	w	–	–
SGU	–	–	–	–	–	w	–	–	–	–	–	w	–	–	–	–	–	*	–	–	–	–	–	w	–
NNA	w	–	–	–	–	–	w	–	–	–	–	–	w	–	–	–	–	–	*	–	–	–	–	–	–
NNU	–	w	–	–	–	–	–	w	–	–	–	–	–	w	–	–	–	–	–	*	–	–	–	–	–
NFA	–	–	w	–	–	–	–	–	w	–	–	–	–	–	w	–	–	–	–	–	*	–	–	–	–
NFU	–	–	–	w	–	–	–	–	–	w	–	–	–	–	–	w	–	–	–	–	–	*	–	–	–
NGA	–	–	–	–	w	–	–	–	–	–	w	–	–	–	–	–	w	–	–	–	–	–	*	–	–
NGU	–	–	–	–	–	w	–	–	–	–	–	w	–	–	–	–	–	w	–	–	–	–	–	*	–
D	–	–	–	–	–	–	–	–	–	–	–	–	–	–	–	–	–	–	–	–	–	–	–	–	*

GEMACO formula: intra+inter.[i+t*x(7)]
Fixed value 0 for intra

3 Transience

	CNA	CNU	CFA	CFU	CGA	CGU	GNA	GNU	GFA	GFU	GGA	GGU	SNA	SNU	SFA	SFU	SGA	SGU	NNA	NNU	NFA	NFU	NGA	NGU	D
CNA	*	–	–	–	–	–	–	–	–	–	–	–	–	–	–	–	–	–	–	–	–	–	–	–	t
CNU	–	*	–	–	–	–	–	–	–	–	–	–	–	–	–	–	–	–	–	–	–	–	–	–	t
CFA	–	–	*	–	–	–	–	–	–	–	–	–	–	–	–	–	–	–	–	–	–	–	–	–	t
CFU	–	–	–	*	–	–	–	–	–	–	–	–	–	–	–	–	–	–	–	–	–	–	–	–	t
CGA	–	–	–	–	*	–	–	–	–	–	–	–	–	–	–	–	–	–	–	–	–	–	–	–	t
CGU	–	–	–	–	–	*	–	–	–	–	–	–	–	–	–	–	–	–	–	–	–	–	–	–	t
GNA	–	–	–	–	–	–	*	–	–	–	–	–	–	–	–	–	–	–	–	–	–	–	–	–	t
GNU	–	–	–	–	–	–	–	*	–	–	–	–	–	–	–	–	–	–	–	–	–	–	–	–	t
GFA	–	–	–	–	–	–	–	–	*	–	–	–	–	–	–	–	–	–	–	–	–	–	–	–	t
GFU	–	–	–	–	–	–	–	–	–	*	–	–	–	–	–	–	–	–	–	–	–	–	–	–	t
GGA	–	–	–	–	–	–	–	–	–	–	*	–	–	–	–	–	–	–	–	–	–	–	–	–	t
GGU	–	–	–	–	–	–	–	–	–	–	–	*	–	–	–	–	–	–	–	–	–	–	–	–	t
SNA	–	–	–	–	–	–	–	–	–	–	–	–	*	–	–	–	–	–	–	–	–	–	–	–	t
SNU	–	–	–	–	–	–	–	–	–	–	–	–	–	*	–	–	–	–	–	–	–	–	–	–	t
SFA	–	–	–	–	–	–	–	–	–	–	–	–	–	–	*	–	–	–	–	–	–	–	–	–	t
SFU	–	–	–	–	–	–	–	–	–	–	–	–	–	–	–	*	–	–	–	–	–	–	–	–	t
SGA	–	–	–	–	–	–	–	–	–	–	–	–	–	–	–	–	*	–	–	–	–	–	–	–	t
SGU	–	–	–	–	–	–	–	–	–	–	–	–	–	–	–	–	–	*	–	–	–	–	–	–	t
NNA	–	–	–	–	–	–	–	–	–	–	–	–	–	–	–	–	–	–	*	–	–	–	–	–	t
NNU	–	–	–	–	–	–	–	–	–	–	–	–	–	–	–	–	–	–	–	*	–	–	–	–	t
NFA	–	–	–	–	–	–	–	–	–	–	–	–	–	–	–	–	–	–	–	–	*	–	–	–	t
NFU	–	–	–	–	–	–	–	–	–	–	–	–	–	–	–	–	–	–	–	–	–	*	–	–	t
NGA	–	–	–	–	–	–	–	–	–	–	–	–	–	–	–	–	–	–	–	–	–	–	*	–	t
NGU	–	–	–	–	–	–	–	–	–	–	–	–	–	–	–	–	–	–	–	–	–	–	–	*	t
D	–	–	–	–	–	–	–	–	–	–	–	–	–	–	–	–	–	–	–	–	–	–	–	–	*

GEMACO formula: i
Fixed value 0 when no transience assumed
In those models assuming transience:
GEMACO formula: intra+inter[a(1 2,3 4]
Fixed value 0 for intra and inter.a(3 4).

4 Survival

	CNA	CNU	CFA	CFU	CGA	CGU	GNA	GNU	GFA	GFU	GGA	GGU	SNA	SNU	SFA	SFU	SGA	SGU	NNA	NNU	NFA	NFU	NGA	NGU	D
CNA	s	–	–	–	–	–	–	–	–	–	–	–	–	–	–	–	–	–	–	–	–	–	–	–	$*$
CNU	–	s	–	–	–	–	–	–	–	–	–	–	–	–	–	–	–	–	–	–	–	–	–	–	$*$
CFA	–	–	s	–	–	–	–	–	–	–	–	–	–	–	–	–	–	–	–	–	–	–	–	–	$*$
CFU	–	–	–	s	–	–	–	–	–	–	–	–	–	–	–	–	–	–	–	–	–	–	–	–	$*$
CGA	–	–	–	–	s	–	–	–	–	–	–	–	–	–	–	–	–	–	–	–	–	–	–	–	$*$
CGU	–	–	–	–	–	s	–	–	–	–	–	–	–	–	–	–	–	–	–	–	–	–	–	–	$*$
GNA	–	–	–	–	–	–	s	–	–	–	–	–	–	–	–	–	–	–	–	–	–	–	–	–	$*$
GNU	–	–	–	–	–	–	–	s	–	–	–	–	–	–	–	–	–	–	–	–	–	–	–	–	$*$
GFA	–	–	–	–	–	–	–	–	s	–	–	–	–	–	–	–	–	–	–	–	–	–	–	–	$*$
GFU	–	–	–	–	–	–	–	–	–	s	–	–	–	–	–	–	–	–	–	–	–	–	–	–	$*$
GGA	–	–	–	–	–	–	–	–	–	–	s	–	–	–	–	–	–	–	–	–	–	–	–	–	$*$
GGU	–	–	–	–	–	–	–	–	–	–	–	s	–	–	–	–	–	–	–	–	–	–	–	–	$*$
SNA	–	–	–	–	–	–	–	–	–	–	–	–	s	–	–	–	–	–	–	–	–	–	–	–	$*$
SNU	–	–	–	–	–	–	–	–	–	–	–	–	–	s	–	–	–	–	–	–	–	–	–	–	$*$
SFA	–	–	–	–	–	–	–	–	–	–	–	–	–	–	s	–	–	–	–	–	–	–	–	–	$*$
SFU	–	–	–	–	–	–	–	–	–	–	–	–	–	–	–	s	–	–	–	–	–	–	–	–	$*$
SGA	–	–	–	–	–	–	–	–	–	–	–	–	–	–	–	–	s	–	–	–	–	–	–	–	$*$
SGU	–	–	–	–	–	–	–	–	–	–	–	–	–	–	–	–	–	s	–	–	–	–	–	–	$*$
NNA	–	–	–	–	–	–	–	–	–	–	–	–	–	–	–	–	–	–	s	–	–	–	–	–	$*$
NNU	–	–	–	–	–	–	–	–	–	–	–	–	–	–	–	–	–	–	–	s	–	–	–	–	$*$
NFA	–	–	–	–	–	–	–	–	–	–	–	–	–	–	–	–	–	–	–	–	s	–	–	–	$*$
NFU	–	–	–	–	–	–	–	–	–	–	–	–	–	–	–	–	–	–	–	–	–	s	–	–	$*$
NGA	–	–	–	–	–	–	–	–	–	–	–	–	–	–	–	–	–	–	–	–	–	–	s	–	$*$
NGU	–	–	–	–	–	–	–	–	–	–	–	–	–	–	–	–	–	–	–	–	–	–	–	s	$*$
D	–	–	–	–	–	–	–	–	–	–	–	–	–	–	–	–	–	–	–	–	–	–	–	–	$*$

GEMACO formula: intra+inter.[[i+t*x(4)]+area]
Fixed value 1 for intra

5 Capture

	CNA	CNU	CFA	CFU	CGA	CGU	GNA	GNU	GFA	GFU	GGA	GGU	SNA	SNU	SFA	SFU	SGA	SGU	NNA	NNU	NFA	NFU	NGA	NGU	D
CNA	p	$*$	–	–	–	–	–	–	–	–	–	–	–	–	–	–	–	–	–	–	–	–	–	–	–
CNU	p	$*$	–	–	–	–	–	–	–	–	–	–	–	–	–	–	–	–	–	–	–	–	–	–	–
CFA	–	–	p	$*$	–	–	–	–	–	–	–	–	–	–	–	–	–	–	–	–	–	–	–	–	–
CFU	–	–	p	$*$	–	–	–	–	–	–	–	–	–	–	–	–	–	–	–	–	–	–	–	–	–
CGA	–	–	–	–	p	$*$	–	–	–	–	–	–	–	–	–	–	–	–	–	–	–	–	–	–	–
CGU	–	–	–	–	p	$*$	–	–	–	–	–	–	–	–	–	–	–	–	–	–	–	–	–	–	–
GNA	–	–	–	–	–	–	p	$*$	–	–	–	–	–	–	–	–	–	–	–	–	–	–	–	–	–
GNU	–	–	–	–	–	–	p	$*$	–	–	–	–	–	–	–	–	–	–	–	–	–	–	–	–	–
GFA	–	–	–	–	–	–	–	–	p	$*$	–	–	–	–	–	–	–	–	–	–	–	–	–	–	–
GFU	–	–	–	–	–	–	–	–	p	$*$	–	–	–	–	–	–	–	–	–	–	–	–	–	–	–
GGA	–	–	–	–	–	–	–	–	–	–	p	$*$	–	–	–	–	–	–	–	–	–	–	–	–	–
GGU	–	–	–	–	–	–	–	–	–	–	p	$*$	–	–	–	–	–	–	–	–	–	–	–	–	–
SNA	–	–	–	–	–	–	–	–	–	–	–	–	p	$*$	–	–	–	–	–	–	–	–	–	–	–
SNU	–	–	–	–	–	–	–	–	–	–	–	–	p	$*$	–	–	–	–	–	–	–	–	–	–	–
SFA	–	–	–	–	–	–	–	–	–	–	–	–	–	–	p	$*$	–	–	–	–	–	–	–	–	–
SFU	–	–	–	–	–	–	–	–	–	–	–	–	–	–	p	$*$	–	–	–	–	–	–	–	–	–
SGA	–	–	–	–	–	–	–	–	–	–	–	–	–	–	–	–	P	$*$	–	–	–	–	–	–	–
SGU	–	–	–	–	–	–	–	–	–	–	–	–	–	–	–	–	P	$*$	–	–	–	–	–	–	–
NNA	–	–	–	–	–	–	–	–	–	–	–	–	–	–	–	–	–	–	p	$*$	–	–	–	–	–
NNU	–	–	–	–	–	–	–	–	–	–	–	–	–	–	–	–	–	–	p	$*$	–	–	–	–	–
NFA	–	–	–	–	–	–	–	–	–	–	–	–	–	–	–	–	–	–	–	–	p	$*$	–	–	–
NFU	–	–	–	–	–	–	–	–	–	–	–	–	–	–	–	–	–	–	–	–	p	$*$	–	–	–
NGA	–	–	–	–	–	–	–	–	–	–	–	–	–	–	–	–	–	–	–	–	–	–	P	$*$	–
NGU	–	–	–	–	–	–	–	–	–	–	–	–	–	–	–	–	–	–	–	–	–	–	P	$*$	–
D	–	–	–	–	–	–	–	–	–	–	–	–	–	–	–	–	–	–	–	–	–	–	–	–	$*$

GEMACO formula: intra.[d+od]+inter.[from(1:2:25,2:2:25)+t]
Fixed values: 1 for intra.d, 0 for intra.od

6 GEOPlacement

	CNA	CNU	CFA	CFU	CGA	CGU	GNA	GNU	GFA	GFU	GGA	GGU	SNA	SNU	SFA	SFU	SGA	SGU	NNA	NNU	NFA	NFU	NGA	NGU	D
CNA	*	–	–	–	g	–	–	–	–	–	–	–	–	–	–	–	–	–	–	–	–	–	–	–	–
CNU	–	*	–	–	–	–	–	–	–	–	–	–	–	–	–	–	–	–	–	–	–	–	–	–	–
CFA	*	–	g	–	g	–	–	–	–	–	–	–	–	–	–	–	–	–	–	–	–	–	–	–	–
CFU	–	–	–	*	–	–	–	–	–	–	–	–	–	–	–	–	–	–	–	–	–	–	–	–	–
CGA	*	–	–	–	g	–	–	–	–	–	–	–	–	–	–	–	–	–	–	–	–	–	–	–	–
CGU	–	–	–	–	–	*	–	–	–	–	–	–	–	–	–	–	–	–	–	–	–	–	–	–	–
GNA	–	–	–	–	–	–	*	–	–	–	g	–	–	–	–	–	–	–	–	–	–	–	–	–	–
GNU	–	–	–	–	–	–	–	*	–	–	–	–	–	–	–	–	–	–	–	–	–	–	–	–	–
GFA	–	–	–	–	–	–	*	–	g	–	g	–	–	–	–	–	–	–	–	–	–	–	–	–	–
GFU	–	–	–	–	–	–	–	–	–	*	–	–	–	–	–	–	–	–	–	–	–	–	–	–	
GGA	–	–	–	–	–	–	*	–	–	–	g	–	–	–	–	–	–	–	–	–	–	–	–	–	–
GGU	–	–	–	–	–	–	–	–	–	–	–	*	–	–	–	–	–	–	–	–	–	–	–	–	–
SNA	–	–	–	–	–	–	–	–	–	–	–	–	*	–	–	–	g	–	–	–	–	–	–	–	–
SNU	–	–	–	–	–	–	–	–	–	–	–	–	–	*	–	–	–	–	–	–	–	–	–	–	–
SFA	–	–	–	–	–	–	–	–	–	–	–	–	*	–	g	–	g	–	–	–	–	–	–	–	–
SFU	–	–	–	–	–	–	–	–	–	–	–	–	–	–	–	*	–	–	–	–	–	–	–	–	–
SGA	–	–	–	–	–	–	–	–	–	–	–	–	*	–	–	–	g	–	–	–	–	–	–	–	–
SGU	–	–	–	–	–	–	–	–	–	–	–	–	–	–	–	–	–	*	–	–	–	–	–	–	–
NNA	–	–	–	–	–	–	–	–	–	–	–	–	–	–	–	–	–	–	*	–	–	–	g	–	–
NNU	–	–	–	–	–	–	–	–	–	–	–	–	–	–	–	–	–	–	–	*	–	–	–	–	–
NFA	–	–	–	–	–	–	–	–	–	–	–	–	–	–	–	–	–	–	*	–	g	–	g	–	–
NFU	–	–	–	–	–	–	–	–	–	–	–	–	–	–	–	–	–	–	–	–	–	*	–	–	–
NGA	–	–	–	–	–	–	–	–	–	–	–	–	–	–	–	–	–	–	*	–	–	–	g	–	–
NGU	–	–	–	–	–	–	–	–	–	–	–	–	–	–	–	–	–	–	–	–	–	–	–	*	–
D	–	–	–	–	–	–	–	–	–	–	–	–	–	–	–	–	–	–	–	–	–	–	–	–	*

formula : intra.[toFA+toGA]+inter.[d+od]
Fixed values: 0 for intra toFA, 1 for inter.d, 0 for inter.od

Observation matrix

	0	1	2	3	4	5	6	7
CNA	−	∗	−	−	−	−	−	−
CNU	∗	−	−	−	−	−	−	−
CFA	−	∗	−	−	−	−	−	−
CFU	∗	−	−	−	−	−	−	−
CGA	−	−	∗	−	−	−	−	−
CGU	∗	−	−	−	−	−	*e*	−
GNA	−	∗	−	−	−	−	−	−
GNU	∗	−	−	−	−	−	−	−
GFA	−	∗	−	−	−	−	−	−
GFU	∗	−	−	−	−	−	−	−
GGA	−	−	∗	*e*	−	−	−	−
GGU	∗	−	−	−	−	−	−	−
SNA	−	∗	−	−	−	−	−	−
SNU	∗	−	−	−	−	−	−	−
SFA	−	∗	−	−	−	−	−	−
SFU	∗	−	−	−	−	−	−	−
SGA	−	−	∗	−	*e*	−	−	−
SGU	∗	−	−	−	−	−	−	−
NNA	−	∗	−	−	−	−	−	−
NNU	∗	−	−	−	−	−	−	−
NFA	−	∗	−	−	−	−	−	−
NFU	∗	−	−	−	−	−	−	−
NGA	−	−	∗	−	−	*e*	−	−
NGU	∗	−	−	−	−	−	−	*e*
D	∗	−	−	−	−	−	−	−

GEMACO formula intrao+intero.to(3 4 5,6,7)
Fixed values: 0 for intra, 1 for inter.to(3 4 5)
