# Supplementary material for: Individual Choices of Wintering Areas Drive Adult Survival Heterogeneity in a Long‐Lived Seabird
Source: Ecol Evol. 2024 Dec 12;14(12):e70675. doi: 10.1002/ece3.70675 (PMC11638144; doi:10.1002/ece3.70675)
Supplement: Supplementary file 2 — Table S1‐S2 [file ECE3-14-e70675-s002.pdf]

Supporting Information S2

Individual choices of wintering areas drive adult survival heterogeneity in a long-lived seabird

Table S1. Model selection (see Methods) for assessing factors affecting survival in Scopoli's shearwater breeding in Pantaleu islet and wintering in different Atlantic wintering areas (Models A and B). All models assumed a temporal recapture probability and differences between animals captured and not captured on the previous occasion (i.e. trap-dependence), Initial State probabilities not varying over time, and time-varying probability of recovering tracking data information. Best models are shown in bold and the models used for estimating annual mean survival are shown in italics. "+" indicates additivity between factors and "*" interaction. np = number of parameters, QAICc = AIC value corrected for overdispersion and sample size, *w* = weight of the model, ΔQAICc = QAICc difference points from the best model.

Model	np	Deviance	QAICc	w	ΔQAICc	IS	Wint	First survival	Adult Survival	Capture
**1**	**35**	**9736.966**	**4359.714**	**0.092**	**0.000**	**Canary,others**	**wNAO**	**Equal**	**SOI+area**	**trap+t**
**2**	**36**	**9733.113**	**4360.033**	**0.078**	**0.320**	**Canary,others**	**wNAO**	**Different**	**SOI+area**	**trap+t**
**3**	**36**	**9733.178**	**4360.062**	**0.077**	**0.349**	**Canary,others**	**wNAO+Canary, others**	**Equal**	**SOI+area**	**trap+t**
**4**	**37**	**9730.212**	**4360.773**	**0.054**	**1.060**	**Canary,others**	**wNAO+Canary, others**	**Different**	**SOI+area**	**trap+t**
5	***34***	***9743.974***	***4360.784***	***0.054***	***1.070***	***Canary,others***	***wNAO***	***Equal***	***area***	***trap+t***
**6**	**37**	**9730.593**	**4360.941**	**0.050**	**1.228**	**Canary,others**	**wNAO * Canary, others**	**Equal**	**SOI+area**	**trap+t**

7	32	9755.559	4361.855	0.031	2.142	Canary,others	Constant	Different	SOI	trap+t
8	*31*	*9760.175*	*4361.874*	*0.031*	*2.160*	*Canary,others*	*Constant*	*Different*	*Constant*	*trap+t*
9	33	9751.351	4362.017	0.029	2.304	Canary,others	wNAO	Different	SOI	trap+t
10	14	9838.142	4362.033	0.029	2.320	Canary,others	wNAO	Equal	SOI+area	trap
11	32	9756.027	4362.062	0.028	2.348	Canary,others	wNAO+area	Different	Constant	trap+t
12	32	9756.027	4362.062	0.028	2.348	Canary,others	wNAO	Different	Constant	trap+t
13	38	9729.057	4362.282	0.025	2.569	Canary,others	wNAO* Canary,Angola,others	Different	SOI+area	trap+t
14	37	9733.643	4362.285	0.025	2.571	Canary,others	wNAO+to Canary, others	Equal	SOI+area	trap+t
15	34	9747.714	4362.431	0.024	2.718	Canary,others	Constant	Equal	SOI+area	trap+t
16	33	9752.340	4362.453	0.023	2.740	Canary,others	Constant	Different	SOI	trap+t
A1	*33*	*9752.432*	*4362.494*	*0.023*	*2.780*	*Canary,others*	*Constant*	*Equal*	*area*	*trap+t*
17	35	9743.541	4362.610	0.022	2.896	Canary,others	Constant	Different	SOI+area	trap+t
18	35	9743.541	4362.610	0.022	2.896	Canary,others	Constant	Different	SOI+area	trap+t
19	34	9748.979	4362.989	0.018	3.275	Canary,others	Constant	Different	area	trap+t
20	35	9744.461	4363.015	0.018	3.302	Canary,others	Canary,others	Equal	SOI+area	trap+t
21	11	9854.064	4363.029	0.018	3.315	Canary,others	Constant	Different	SOI	trap
22	33	9753.695	4363.050	0.017	3.337	Canary,others	Constant	Different	Canary,Angola,others	trap+t
23	36	9740.375	4363.233	0.016	3.519	Canary,others	Constant	SOI	SOI+area	trap+t
24	36	9740.379	4363.234	0.016	3.521	Canary,others	Canary,others	Different	SOI+area	trap+t
25	38	9732.438	4363.772	0.014	3.710	area	wNAO	Different	SOI+area	trap+t
26	32	9759.154	4363.439	0.014	3.726	Canary,others	Constant	Different	GEO	trap+t
27	37	9736.953	4363.743	0.012	4.029	Canary,others	Constant	Equal	SOI*area	trap+t
28	14	9842.164	4363.805	0.012	4.092	Canary,others	Constant	Different	SOI+area	trap
29	*33*	*9755.528*	*4363.858*	*0.012*	*4.144*	*area*	*Constant*	*Different*	*Constant*	*trap+t*
30	36	9742.767	4364.286	0.011	4.224	area	wNAO	Equal	area	trap+t
31	37	9737.484	4363.977	0.011	4.263	Canary,others	wNAO*others+Canary	Different	SOI+area	trap+t
32	38	9733.500	4364.240	0.010	4.526	Canary,others	Constant	Different	SOI*area	trap+t
33	35	9748.638	4364.855	0.007	5.142	Canary,others	Constant	SOI	area	trap+t

34	38	9735.221	4364.998	0.007	5.284	Canary,others	Constant	Different	wNAO	trap+t
35	14	9845.071	4365.086	0.006	5.372	Canary,others	Canary, Angola, others	wNAO	area	
36	14	9846.369	4365.657	0.005	5.944	Canary,others	Constant	Different	area	trap
37	34	9755.455	4365.842	0.004	6.128	Canary,others	area	Equal	area	trap+t
A2	35	9751.162	4365.967	0.004	6.254	area	Constant	Equal	area	trap+t
38	35	9751.526	4366.128	0.004	6.414	area	Constant	Different	Canary,Angola,others	trap+t
39	38	9739.138	4366.724	0.003	7.010	area	Canary,Angola,others	Different	area	trap+t
40	36	9748.391	4366.764	0.003	7.050	area	Constant	Different	area	trap+t
41	41	9726.291	4367.122	0.002	7.408	Canary,others	Canary,others	Different	SOI*area	trap+t
42	37	9744.975	4367.277	0.002	7.563	area	Canary,others	Different	area	trap+t
43	40	9732.971	4368.045	0.001	8.331	Canary,others	Constant	Different	SOI*area	trap+t
44	39	9737.558	4368.046	0.001	8.332	area	area	Different	area	trap+t
45	38	9743.179	4368.504	0.001	8.790	Canary,others	First breeders	area	SOI	trap+t
A3	*30*	*9781.162*	*4369.104*	*0.001*	*9.390*	*Canary,others*	*Constant*	*Equal*	*Constant*	*trap+t*
46	33	9770.622	4370.507	0.000	10.793	Canary,others	First breeders* Canary,others	Equal	SOI+area	trap+t
A4	32	9776.548	4371.101	0.000	11.388	area	Constant	Equal	Constant	trap+t
47	32	9777.760	4371.635	0.000	11.922	Canary,others	First breeders	Equal	SOI+area	trap+t
48	39	9747.208	4372.297	0.000	12.584	area	to area	Different	area	trap+t
49	38	9753.833	4373.197	0.000	13.484	Canary,others	Constant	wNAO	SOI + area	trap+t
50	38	9755.061	4373.738	0.000	14.025	Canary,others	Constant	SOI*area	Constant	trap+t
51	29	9799.377	4375.114	0.000	15.400	Equal	Constant	Equal	Constant	trap+t
52	51	9733.572	4390.552	0.000	30.839	Canary,others	Constant	Equal	t	trap+t
53	47	9769.140	4398.126	0.000	38.412	Canary,others	t	Equal	SOI	trap+t
54	97	9749.034	4491.017	0.000	131.304	Canary,others	Constant	Different	area*t	trap+t

Table S2. Short- and long-term effects of the logger deployment on adult survival. a) Model selection for assessing short-term effects of the logger deployment on adult survival, by capture–mark–recapture modelling and b) estimates of annual survival for those individuals with geolocator (GEO) and those without geolocator (NO GEO) from models 26, G1 and G4 (Table S1).

a)

Model	1st Survival	Survival	np	Deviance	QAICc	AICcW	ΔAIC
**7**	**Different**	**SOI**	**32**	**9755,56**	**4361,86**	**0,34**	**0,00**
**8**	**Different**	**constant**	**31**	**9760,175**	**4361,874**	**0,34**	**0,02**
**26**	**Different**	**GEO**	**32**	**9759,154**	**4363,439**	**0,16**	**1,58**
G1	GEO	GEO	33	9758,93	4365,35	0,06	3,50
G2	GEO	SOI	34	9755,88	4366,03	0,04	4,17
G3	GEO	constant	33	9760,58	4366,08	0,04	4,23
A3	Equal	constant	30	9781,16	4369,10	0,01	7,25
G4	Equal	GEO	31	9780,88	4370,99	0,00	9,14
G5	Equal	GEO+SOI	32	9777,89	4371,69	0,00	9,84
G5	Different	GEO*SOI	33	9777,80	4373,67	0,00	11,81

b)

Model		1stMortality	Survival (not 1st year)
26	NO GEO	0.090 (0.047-0.166)	0.843 (0.822-0.862)
	GEO		0.814 (0.734-0.875)
G1	NO GEO	0.092 (0.048-0.169)	0.843 (0.823-0.862)
	GEO	0.041 (0.000-0.988)	0.811 (0.723-0.875)
	NO GEO	-	0.828 (0.809-0.845)
G4	GEO		0.808 (0.728-0.868)
